# Nonlinear and Spatial Effects of Tourism on Carbon Emissions in China: A Spatial Econometric Approach

**DOI:** 10.3390/ijerph16183353

**Published:** 2019-09-11

**Authors:** Chao Bi, Jingjing Zeng

**Affiliations:** 1 International Business School, Shaanxi Normal University, Xi’an 710119, China; bichao@snnu.edu.cn; 2 School of Public Administration, Zhongnan University of Economics and Law, Wuhan 430073, China

**Keywords:** nonlinear effects, spatial lag effects, tourism industry, carbon emissions, spatial econometric approach

## Abstract

Reducing carbon emissions is crucial to the sustainable development of tourism. However, there are no consistent conclusions about the nexus between tourism and carbon emissions. Considering the possible nonlinear and spatial effects of tourism on carbon emissions, this paper employed spatial econometric models combined with quadratic terms of explanatory variables to explore the nexus between them using Chinese provincial panel data from 2003 to 2016. The main results are as follows: (1) There is a significant inverse U-shaped relationship between tourism development and carbon emissions. In the provinces whose tourism receipts are relatively low, the effects of tourism on carbon emissions are positive but decrease gradually as the tourism receipts increase and then shifts to negative and continues decreasing gradually when the tourism receipts beyond the critical value. (2) For the geographical proximity and industrial relevance, one province’s tourism development not only affects its carbon emissions but also affects its neighbors’ carbon emissions through spatial lag effect (indirect effect) which is also inverse U-shaped. (3) Carbon reduction policies, sustainable education, and transportation infrastructure all have significant moderating effects on the relationship between tourism and carbon emissions, but the moderating effect of the management efficiency of tourism is not statistically significant. Furthermore, improvements to the sustainable education and transportation infrastructure not only strengthen the direct negative effect of tourism on carbon emissions but also strengthen the indirect negative effect of tourism on carbon emissions. This study not only advances the existing literature but is also of considerable interest to policymakers.

## 1. Introduction

Tourism is highly vulnerable to climate change, in addition to contributing to it. Threats for the sector are diverse, including various impacts such as extreme weather events, increasing insurance cost and safety concerns, biodiversity loss, and so on. At the same time, tourism is one of the key drivers to the anthropogenic component of climate change [1,2], which is predicted to contribute approximately 7.5% of global carbon emissions in 2035 [3]. Therefore, reducing carbon emissions from tourism not only helps to offset global warming but is also conducive to the sustainable development of the tourism industry. The effective way of reducing carbon emissions is dependent on the linkage between tourism and carbon emissions. Although the nexus between tourism and carbon emissions has been widely studied over time, a lack of tourism statistics and materials makes it difficult to quantify carbon emissions from tourism [4]. Tourism is not a traditional sector in the System of National Accounts, and as a result, the statistics of carbon emissions of the tourism industry on a national or regional scale is difficult to calculate. Furthermore, it is also challenging to assess the other two kinds of carbon emission effects of tourism: Income effect and infrastructure effect. The income effect means that tourism growth is helpful in increasing residents’ income, and then affecting carbon emissions, because existing studies have already confirmed that per capita income usually has a significant impact on carbon emissions [5,6]. The infrastructure effect means that the development of tourism usually requires a large amount of infrastructure investment, which affect carbon emissions, because the investment on tourism infrastructure (e.g., transportation infrastructure, information infrastructure, and building infrastructure) usually has a significant impact on carbon emissions [7]. Therefore, a credible evaluation of the aggregated effects of tourism on carbon emissions will improve the ability to manage the sustainable development of tourism.

Research on the carbon emissions of the tourism industry has been widely carried out and discussed, although it has been difficult to measure the carbon emissions from tourism for many years. Existing studies can be divided into two categories based on the data used in the research. The first category of the research has mainly focused on the calculation of carbon emissions from tourism using methods such as a bottom–up approach, top–down approach, and a carbon footprint approach [4], and then they assessed the relationship between tourism development and carbon emissions with the calculated emissions data [8,9,10]. Although this kind of literature has shed some light on the relationship between tourism and carbon emissions, ignoring the income effect and infrastructure effect of tourism mentioned above may cause inaccurate results [11,12]. The second category of the literature used econometric models with statistical data of national or regional carbon emissions to estimate the effects of tourism on carbon emissions [13]. As the statistical data of carbon emissions contain the income effect and infrastructure effect of tourism, the overall effect of tourism on carbon emissions can be estimated easily using this kind of data. However, the latter kind of literature still has some limitations, which may raise questions regarding the robustness and validity of the findings. Firstly, these studies typically explored the impact of tourism on carbon emissions based on linear regression models, and few studies have focused on the nonlinear connection between tourism and carbon emissions. Secondly, this literature has not accounted for the spatial dependence of different regions, while a region’s carbon emissions and tourism development are usually related to those of its neighbors [14,15].

This paper aims to address the gaps by modeling the effects of tourism development on carbon emissions in the context of spatial dependence and nonlinear impact using the panel data of 30 provinces in China from 2003 to 2016. There are two reasons to choose China’s provinces as the research samples in this study. To begin with, China’s tourism industry has developed rapidly in recent years, and the induced environmental impacts are getting more and more attention [16]. However, the studies on the overall effect of tourism on carbon emissions are relatively scarce. Additionally, panel data of China’s 30 provinces from 2003 to 2016 provide the possibility to study the complicated relationship between tourism development and carbon emissions. The main contributions of this study are threefold. Firstly, this study employed a quadratic polynomial model to test the nonlinear relationship between tourism and carbon emissions. Secondly, a panel spatial econometric technique was used to take spatial dependence of carbon emissions into consideration. Finally, the moderating effects of the variables that affect the carbon efficiency of tourism subsectors were estimated to explore the factors which affect the relationship between tourism and carbon emissions.

This paper is structured as follows: Following the introduction, the next section reviews the recent literature on the calculation of the emissions from the tourism industry and the estimation of the effects of tourism on carbon emissions. The third section introduces model specification, variables, and the data description. Section 4 presents the results and discussion. Finally, we summarize the overall conclusions and policy implications.

## 2. Literature Review

### 2.1. Calculation of the Emissions from the Tourism Industry

Due to the lack of the census data on the carbon emissions from the tourism industry, scholars usually measure carbon emissions data on tourism in a specific country, region or scenic spot as the first step and then further evaluate the dynamic relationship between tourism development and carbon emissions. For example, Becken et al. studied the carbon emissions of ecological hotels in the Lamington National Park. The hotels had been granted the Green Globe 21 Certificate. The study showed that after being certified, the hotels reduced carbon dioxide emissions by 189 tons per year [8]. In another study, Wu and Shi estimated carbon emissions from China’s tourism sector in 2008. According to their estimation, carbon emissions from tourism sector amounted to 51.34 million tons, accounting for 0.86% of the total in China [9]. In a study by Xie et al., they measured the carbon emissions from the tourism of the Yangtze River Delta area. The results showed that there is a positive relationship between carbon emissions from tourism and the gross income of tourism [10]. Finally, Wu et al. calculated the emissions from the tourism of five provinces in China from 2009 to 2011. The main finding of the study was that Beijing and Hainan saw their emissions per tourist dropped continuously during 2009 and 2011. Zhejiang’s emissions from tourism showed a reverse U-shape trend, while those of Shandong and Hubei showed U-shape trends [4].

Among the above studies, methods of calculating the emissions from tourism have been one of the main concerns. Since the first measurement proposed by Gossling [17], a variety of methods have been explored, integrated, and applied on varied scales from national level down to local [18,19]. There are three kinds of common methods used in the literature: A top–down method [20,21,22,23,24], a bottom–up method [9,10,25,26,27,28], and a combination of other methods (e.g., carbon footprint, life cycle assessment, and environmental satellite accounts) [8,11,12,29,30,31]. Each of these approaches has its advantages. However, there are still some limitations when these methods are used to assess the relationship between carbon emissions and tourism. Firstly, this literature has ignored the additional emission effects which can range from 30% to 110% of the basic effect [12]. Ignoring these effects would substantially underestimate the overall emission effect of tourism consumption [11]. Secondly, one of the key assumptions in these methods is the linearity between expenditure and emissions, implying that the influence of technological progress and management efficiency is not considered [21]. Thirdly, most of the literature typically provides a snapshot of the relationship between tourism and carbon emissions. Long-term evaluations of tourism emissions are scarce, so the environmental improvement or operational improvement of tourism services could not be identified [1].

### 2.2. Estimation of the Comprehensive Effect of Tourism on Carbon Emissions

In recent years, some scholars have attempted to use econometric techniques with the aggregated longitudinal carbon emissions data (e.g., total national carbon emissions and total regional carbon emissions), which include all types of emission effects of the tourism industry [13], to evaluate the long-term comprehensive effects of tourism on carbon emissions. The literature can be divided into two categories based on the type of conclusions. The first kind of literature concluded that tourism has a significant positive impact on carbon emissions. For example, Katircioglu et al. found that for a small island like Cyprus, international tourism arrivals have a significant positive impact on carbon emissions [32]. Katircioglu investigated the long-run equilibrium relationship between tourism and environmental degradation as proxied by carbon emissions in Turkey. The findings reveal that tourism development has resulted in considerable carbon emissions [33]. Using the generalized method of moments model from panel data in 1998–2006, Leon et al. also confirmed the same findings in the context of both developed and less developed countries across the world [34]. In another study, Durbarry and Seetanah explored the dynamic relationship between tourism development and carbon emissions in the case of Mauritius from the period of 1978–2011 using the autoregressive distributed lag (ARDL) approach. The study also provided empirical evidence that an increase in the number of tourists has a considerable and positive impact on carbon emissions [35]. Similar evidence was provided by Zaman et al. and Paramati et al. for developed and developing countries [6,36].

The second kind of literature concluded that the development of tourism has a significant adverse effect on carbon emissions. For example, Lee and Brahmasrene investigated the influence of tourism on carbon emissions using panel data of European Union countries from 1988 to 2009. Results from panel cointegration techniques and fixed-effects models indicated that tourism is inversely related to carbon emissions in the EU [37]. In a different research study, Katircioglu found that tourist arrivals have negatively significant effects on carbon dioxide emission levels both in the long-term and the short-term periods in Singapore [5]. In Raza et al.’s study, they examined the relationship between tourism and carbon emissions using US data. The findings of their study confirmed that tourism development can affect carbon emissions adversely [38]. Finally, using panel data of Western European Union countries, Paramati et al. also found that the expansion of the tourism industry can decrease carbon emissions [39].

According to the above conclusions, although most of the studies confirm the existence of an empirical relationship between carbon emissions and tourism development, the direction of causality between them remains unclear. One of the main reasons for the inconsistent conclusions may be because they ignored the nonlinear effect of tourism on carbon emissions [40,41]. Additionally, ignoring the spatial dependence among the regions may also cause inaccurate conclusions [14,42,43,44]. Therefore, with the end of taking both the spatial dependence and nonlinearity into consideration, this study employs a panel spatial econometric model containing a quadratic polynomial relationship to estimate the total effects of tourism on carbon emissions.

## 3. Model Specification, Variables, and Data Description

### 3.1. Model Specification

#### 3.1.1. Modeling of the Nonlinearity

This paper argues that there is an inverse U-shaped relationship between tourism and carbon emissions for the following two reasons. Firstly, tourism growth will increase carbon emissions. Tourism depends on a wide range of infrastructure services such as airports, ports, roads, railheads, resorts, and restaurants, as well as telecommunications and so on. Building the above ancillary infrastructure of tourism generates massive carbon emissions [45,46,47,48]. Furthermore, the transportation and hosting of increasing tourism consumers also induce more and more energy consumption and carbon emissions. Secondly, well-managed tourism can play a positive role in the environment [3]. With the growth of tourism, management efficiency of enterprises in the tourism subsectors will be improved for the learning-by-doing effect [49]. Improved management leads to better fuel efficiency, lower energy intake per unit operation, and subsequently lower emission levels [50]. Further, carbon emissions can be reduced through technological progress and adopting clean energy, all of which will be increased by the development of tourism [51].

In order to reveal the above nonlinear influence of tourism on carbon emissions, the model is preliminarily set as follows:

(1) $$Emission_{it}=\beta_{0}+\beta_{1}Tourism_{it}+\beta_{2}Tourism_{it}^{2}+\beta_{k}Controls+u_{i}+v_{t}+\varepsilon_{it}$$

where *i* and *t* represent region and year respectively; *Emission* is the carbon emission; *Tourism* is the development of tourism; *Controls* represents a series of control variables; *u_i_* and *v_t_* represent regional fixed effect and time fixed effect respectively; ε is the error term; *β*_0_ is the constant item; and *β*_1_, *β*_2_, and *β*_k_ are the coefficients to be estimated.

#### 3.1.2. Modeling of the Spatial Dependence

There are usually similar economic structure and living customs among neighboring provinces so that their energy consumption and induced carbon emissions are also correlated with each other [52]. Furthermore, the economic development and induced carbon emissions of one province usually can increase the same for its neighbors due to their close economic connections [53]. Additionally, tourism has spatial effects in terms of carbon emissions, because the tourism growth of one province will cause the growth of related industries and the induced carbon emissions of adjacent provinces [14]. To consider the above spatial effects, we can add the spatial relationships into Equation (1) by using a spatial Durbin model (SDM) as follows:

(2) $$\begin{array}{l} Emission_{it}=\beta_{0}+\rho \sum_{j=1}^{n}w_{ij}Emission_{jt}+\beta_{1}Tourism_{it}+\beta_{2}Tourism_{it}^{2}+\beta_{3}\sum_{j=1}^{n}w_{ij}Tourism_{jt}\\ \;+\beta_{4}\sum_{j=1}^{n}w_{ij}Tourism_{jt}^{2}+\beta_{k}Controls+u_{i}+v_{t}+\varepsilon_{it} \end{array}$$

In Equation (2), *ρ* denotes the regression coefficient of spatial lag of the explained variable, that is, the specific province’s carbon emission effect caused by its neighboring provinces; *β*_3_ and *β*_4_ denote the regression coefficients of spatial lag of tourism and its quadratic form respectively, that is, the specific province’s carbon emission effect caused by its neighbors’ tourism growth; *w_ij_* denotes the spatial relationship between province *i*, and province *j* and is defined as follows:

(3) $$w_{ij}=\left\{ \begin{array}{l} 1,\;if\;province\;i\;and\;province\;j\;are\;adjacent;\\ 0,\;other\;situations. \end{array}\right.$$

Although Equation (2) can describe the spatial dependence of carbon emissions, the spatial dependence may be caused by the spatial dependence of omitted unobservable variables (e.g., climate environment shared by neighboring provinces), which are included in the error term. Therefore, we can use the spatial Durbin error model (SDEM) alternatively as follows:

(4) $$\begin{array}{l} Emission_{it}=\beta_{0}+\beta_{1}Tourism_{it}+\beta_{2}Tourism_{it}^{2}+\beta_{3}\sum_{j=1}^{n}w_{ij}Tourism_{jt}\\ \;+\beta_{4}\sum_{j=1}^{n}w_{ij}Tourism_{jt}^{2}+\beta_{k}Controls+u_{i}+v_{t}+u_{it}\\ u_{it}=\lambda \sum_{j=1}^{n}w_{ij}u_{jt}+\varepsilon_{it} \end{array}$$

where *u* denotes the error term containing the spatial dependence, and *λ* denotes the regression coefficient of spatial impacts of the error terms.

To determine which model is more reliable, this paper used the likely ratio test (LR) with a general nested spatial model (GNSM), which can be reduced to SDM or SDEM. The GNSM is defined as follows:

(5) $$\begin{array}{l} Emission_{it}=\beta_{0}+\rho \sum_{j=1}^{n}w_{ij}Emission_{jt}+\beta_{1}Tourism_{it}+\beta_{2}Tourism_{it}^{2}+\beta_{3}\sum_{j=1}^{n}w_{ij}Tourism_{jt}\\ \;+\beta_{4}\sum_{j=1}^{n}w_{ij}Tourism_{jt}^{2}+\beta_{k}Controls+u_{i}+v_{t}+u_{it}\\ u_{it}=\lambda \sum_{j=1}^{n}w_{ij}u_{jt}+\varepsilon_{it} \end{array}$$

#### 3.1.3. Modeling of the Moderating Effects

The tourism industry consists of many subsectors such as transportation, accommodation, and reaction. Therefore, the variables which affect carbon emissions and carbon efficiency of these subsectors will impact the strength of the relationship between the tourism industry and the aggregated carbon emissions. This kind of impact is called moderating effects and can be modeled through the interaction of these variables with the explanatory variables.

### 3.2. Variables

In this paper, carbon emissions are considered as the explained variable, which is denoted by the “emission” and measured using the amount of provincial total carbon emissions; tourism growth is the explanatory variable and measured by the provincial tourism receipts. According to the existing literature, energy consumption, energy mix, and gross domestic production (GDP) per capita are used as the control variables in this paper [54,55,56,57,58].

In terms of the moderating variables, two kinds of variables are taken into consideration. The first kind of moderating variable refers to the variables that affect the carbon efficiency of all the tourism subsectors. Carbon reduction policy, sustainable education, and tourism efficiency [59,60,61,62,63] are three such moderating variables used in this paper. Carbon reduction policy refers to policies to promote the carbon emission abatement and is denoted by “Reduction”, which is measured through the number of provincial carbon abatement policies. Sustainable education is denoted by “Education” and is measured through the average years of schooling. Tourism efficiency is denoted by “Toueff” and is measured using the ratio of tourism receipts to the number of employees. The second kind of moderating variables includes the variables that affect the carbon efficiency of one specific tourism subsector, such as transportation infrastructure, which has a significant impact on the energy efficiency and the induced carbon efficiency of the transportation industry [7]. Because the transportation industry contributes approximately 75% of the carbon emissions from tourism, this paper mainly explored the moderating effects of the transportation infrastructure, which is measured by the intensity of the road networks [12,64] and is denoted by “Trans”.

### 3.3. Data Description

The panel dataset is yearly and covers the period from 2001 to 2016 for 30 Chinese provincial regions. Tibet, Hong Kong, Macau, and Taiwan are excluded due to data constraints. The data of the provincial carbon emissions were taken from Shan et al. [65]; the data of provincial tourism receipts, GDP, population, average years of schooling, and the number of employees of the tourism industry in 30 provinces were taken from the China Statistical Yearbook [66]. Data on energy consumption and energy mix of the 30 provinces were taken from the China Energy Yearbook [67]. Data on carbon reduction policy were taken from Zeng et al. [59]. Data on transportation infrastructure were taken from Bi et al. [7]. Table 1 reports the description and summary statistics for all variables.

## 4. Results and Discussion

### 4.1. Test of the Spatial Dependence of Carbon Emissions

To provide specific insight into the spatial pattern of carbon emissions, we used a visualization technique to describe the spatial distribution of carbon emissions of China in 2003, 2008, 2012, and 2016. The distribution maps are shown as follows.

**Figure 1:**
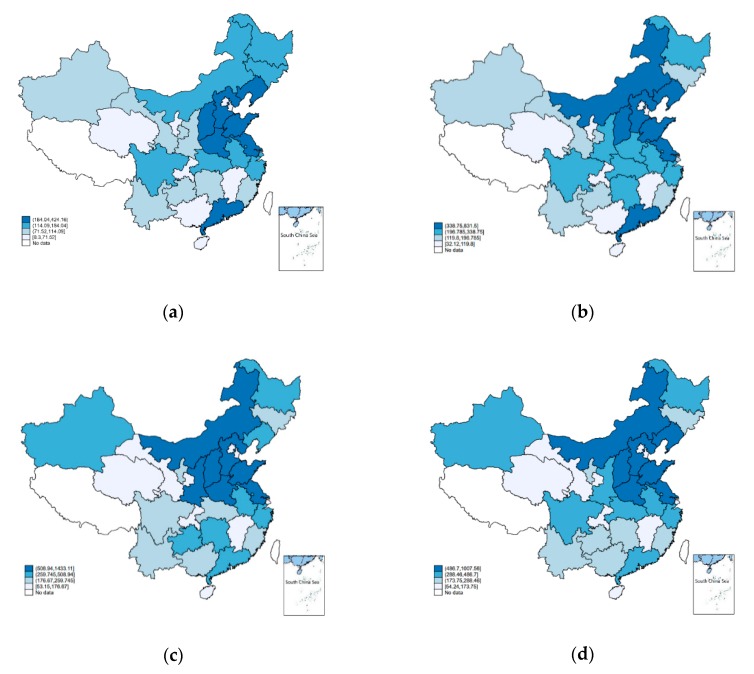
Variation trend of spatial distribution of carbon emissions of China in (**a**) 2003, (**b**) 2008, (**c**) 2012, and (**d**) 2016.

Figure 1 indicates that the provinces with high carbon emissions tend to cluster together with those with also high carbon emissions. In turn, provinces with low carbon emissions tend to cluster with those with low carbon emissions. The above characteristic means that the distribution of carbon emissions in Chinese provinces is spatially dependent on each other. However, it seems that the spatial dependence of provinces with low carbon emissions in 2016 is not significant, so we further calculated Moran’s I statistic of carbon emissions from 2003 to 2016. The results presented in Table 2 shows that there is significant spatial dependence of carbon emissions at the 10% significance level in all the years except 2006, 2007, and 2008. Therefore, it is necessary to consider the spatial dependence using the spatial econometric model.

### 4.2. Estimation Results of the Impact of Tourism on Carbon Emissions

In order to verify the effectiveness of the SDEM model, we simultaneously estimated the SDM model and the GNSM model for comparison. For the estimation of these models, we used the maximum likelihood method proposed by Elhorst [68]. Because the SDM model and the SDEM model are both nested in the GNSM model, we compared these three models using the likelihood ratio test (LR test). To demonstrate the superiority of the spatial econometric model, we also estimated the ordinary panel model (OPM) using the ordinary least square method. The whole process was calculated by Stata 15.1, and the results are shown in Table 3.

The results of the LR test show that the GNSM model is better than the SDM model (the LR value was 0.19 and not significant at the 10% significance level), while the SDEM model is better than the GNSM model (the LR value was 11.18 and significant at the 1% significance level). Therefore, the SDEM model is the best model among these three models. Furthermore, the coefficients of the spatial lags of the error term (W*u) and the spatial lags of tourism and squared tourism (W*tourism and W* tourism^2^) in the SDEM model are all significant with at least a 5% significance level, which means that the spatial dependence should be considered, and the spatial model is better than the ordinary panel model.

According to the results of the SDEM model presented in Table 3, the coefficient of tourism is 3.2639 with a significance level of 10%, and the coefficient of squared tourism is −0.0577, with a significance level of 1%. The positive effect of tourism and the negative effect of squared tourism show that there is an inverse U-shaped relationship between tourism and carbon emissions. The inverse U-shaped relationship implies that one province’s carbon emissions will increase with its tourism growth, but a threshold will eventually be reached, after which the carbon emissions will decrease. The above effect of tourism is also called a direct effect in the context of spatial econometrics. At first glance, this finding is different from the existing literature. However, if the samples of this study are divided into two types, one that includes only the samples whose tourism receipts are on the left of the axis of symmetry of the inverse U-shape, and the other one that includes the samples whose tourism receipts are on the right of the axis of symmetry, the result of the first type is consistent with the literature which found that tourism has a significant positive emissions effect in the countries (e.g., Cyprus, Turkey, and Mauritius) where tourism receipts are relatively low [32,33,34,35,36]. The result of the second type is consistent with the literature which found that tourism has a significant negative emissions effect in the countries (e.g., United States, Western European Countries, and Singapore) where tourism receipts are relatively high [5,37,39]. Therefore, the nonlinear model used in this paper can include both positive and negative effects in one model and is more realistic and appropriate than the existing linear models.

Among the estimation results of the SDEM model in Table 3, the coefficient of the spatial lag of tourism is 7.8268, with a significance level of 5%, and the coefficient of the spatial lag of squared tourism is −0.0899, with a significance level of 1%. The results show that one province’s tourism not only affects its carbon emissions but also affects its neighbors’ carbon emissions through the spatial lag effect, which is also called an indirect effect in the context of spatial econometrics. Although the indirect effect of tourism on carbon emissions turned out to be significant in this paper, it was rarely considered in the existing studies. The geographic proximity of provinces and the mobility of economic resources (e.g., services, products, technologies, and funds) between provinces have increased the transfer of carbon emissions among provinces [53]. From the perspective of tourism development, one province’s tourism growth not only stimulates the growth of the tourism subsectors of the province but also moves the related industry resources across the province. Thus, one province’s tourism-related industry is driven by its neighbors’ tourism development, accompanied by carbon emissions generated during the process of undertaking industry growth. Therefore, one province will bear some carbon emissions for its neighbors’ tourism development and further expand the degree of interprovincial carbon emissions [69,70]. Moreover, the negative coefficient of the spatial lag of squared tourism and the positive coefficient of the spatial lag of tourism mean that there is a nonlinear relationship between tourism and its indirect effect on carbon emissions. When one province’s tourism receipts beyond the critical value, tourism development will not only eliminate carbon emissions of the province but also reduce the carbon emissions of its neighbors.

**Figure 2:**
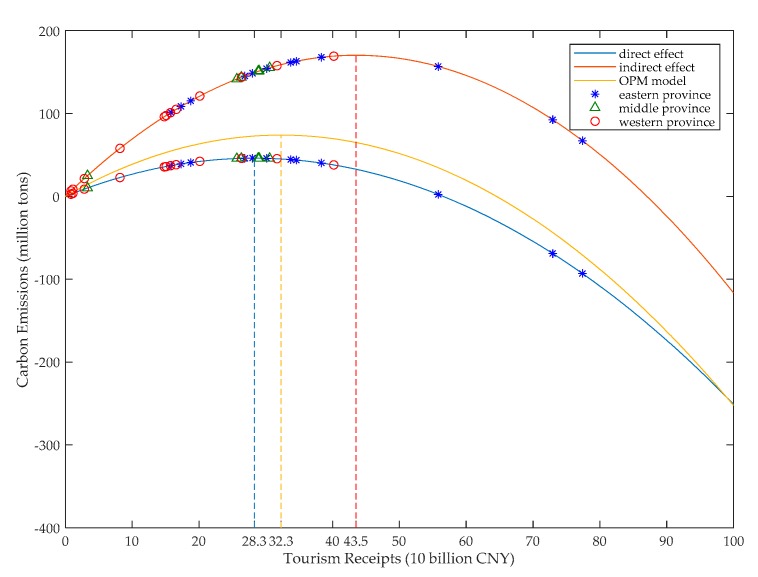
The nonlinear direct and indirect effects of tourism on carbon emissions.

In order to describe the above nonlinear spatial relationships more clearly, we depicted the direct and the indirect effects of tourism on carbon emissions in Figure 2. Additionally, the relationship of tourism on carbon emissions based on the OPM model is also depicted in Figure 2 for comparison. 

Figure 2 illustrates the inverse U-shaped direct effect and indirect effect of tourism on carbon emissions more intuitively. The axis of symmetry of the inverse U-shaped curve is also depicted in Figure 2. According to the axis of symmetry, we can easily determine that when tourism receipts are lower than the critical value (283 billion Chinese Yuan (CNY)), the direct effect of tourism on carbon emissions is positive and then shifts to negative when the tourism receipts are beyond the above critical value. In terms of indirect effect, when the tourism receipts are lower than 435 billion CNY, the effect of tourism on carbon emissions is positive and then shifts to negative when the tourism receipts increase beyond the critical value (435 billion CNY under this situation). Moreover, compared to the SDEM model used in this paper, the inverse U-shape curve which describes the result of the OPM model shows that the nonlinear OPM model overestimates the positive impact on carbon emissions from tourism and underestimates the negative impact on carbon emissions from tourism by ignoring of the abovementioned indirect effect.

The direct and indirect effects of tourism on carbon emissions of 30 provinces in 2016 are also shown in Figure 2. The provinces are divided into eastern provinces, which include Beijing, Tianjin, Hebei, Liaoning, Shanghai, Jiangsu, Zhejiang, Fujian, Shandong, Guangdong, and Hainan, middle provinces which include Jilin, Heilongjiang, Shanxi, Henan, Anhui, Jiangxi, Hubei, and Hunan, and western provinces which include Inner Mongolia, Qinghai, Ningxia, Shaanxi, Sichuan, Chongqing, Guizhou, Yunnan, Guangxi, Gansu, and Xinjiang. According to Figure 2, in terms of the direct effect, there are 12 provinces whose tourism has already exceeded the critical value and has a negative impact on carbon emissions, and the tourism of the rest of the provinces still has a positive effect on carbon emissions. Because most of the above 12 provinces are the relatively developed provinces and most of the remaining provinces are less developed provinces in China, Figure 2 provides further evidence that the relationship between tourism and carbon emissions are consistent with the existing literature [32,33,34,35,36,37,38,39]. In terms of the indirect effect, there are only three provinces whose tourism receipts have exceeded the critical value, which means that most of the provinces in China still have a positive effect on their neighbors’ carbon emissions.

### 4.3. Estimation Results of the Moderating Effects

The SDEM model was used to estimate the moderating effects of reduction policy, sustainable education, tourism efficiency, and transportation infrastructure. The LR test was used to test whether the SDEM model should be reduced to the spatial error model (SEM), which does not contain the spatial lags of explanatory variables compared to the SDEM model. The results are presented in Table 4. 

According to the values of the LR test in Table 4, the SDEM model should be reduced to the SEM model when reduction policy and tourism efficiency are used as moderators, which implies that both the reduction policy and tourism efficiency cannot affect the indirect effect of tourism on carbon emissions.

**Figure 3:**
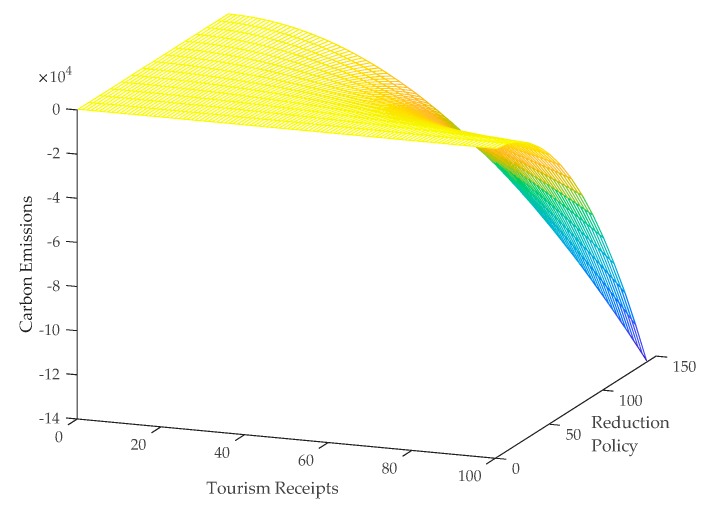
Effect of tourism on carbon emissions using reduction policy as the moderator.

The results in column 3 in Table 4 show that the interaction term of the squared tourism and the reduction policy has a significant negative impact on carbon emissions, which indicates that as the number of the reduction policy increases, the direct negative effect of tourism on carbon emissions tends to be strengthened. Zeng et al. found that the reduction policy can significantly affect the energy efficiency and induced carbon emissions of provincial industries [59]. The findings of this study further confirm that the reduction policy has a significant negative effect on carbon emissions by affecting the carbon efficiency of tourism subsectors. Based on the regression coefficients of the explanatory variables in column 3 of Table 4, this paper depicts the relationship between tourism, reduction policy, and carbon emissions in Figure 3. As shown in Figure 3, we can see that as the number of the reduction policy increases, the same tourism receipts will induce more carbon emission reductions.

**Figure 4:**
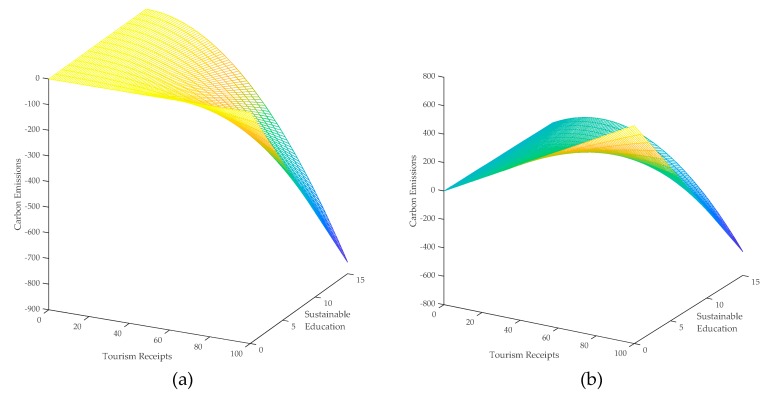
(**a**) Direct and (**b**) indirect effect of tourism on carbon emissions using sustainable education as the moderator.

The results in column 4 in Table 4 show that the interaction term of the squared tourism and education has a significant negative effect on carbon emissions, which implies that the increase of the sustainable education tends to strengthen the negative effect of tourism on carbon emissions. Some scholars argue that the environmental protection awareness of tourists has an essential impact on the carbon emissions induced from tourism [62], and this finding provides empirical evidence for the above argument. The coefficient of the spatial lag of the interaction term is −0.0091 with a significance level of 1%, which indicates that the sustainable education not only strengthens the negative direct effect of tourism on carbon emissions but also strengthens the negative indirect effect of tourism on carbon emissions. The direct and indirect effects of tourism on carbon emissions using sustainable education as the moderator are depicted in Figure 4, which shows the above findings more clearly. 

According to the results in column 7 in Table 4, the coefficient of the squared tourism with the moderator is −0.0192, but it is not statistically significant, which means that the management efficiency of the tourism industry cannot affect the direct effect of tourism on carbon emissions. This finding is different from the arguments of the existing literature [49]. Therefore, although in theory, well-managed tourism could improve energy efficiency and the induced carbon efficiency [3,63], our above results could not provide the empirical evidence for this conclusion using Chinese samples, which indicates that with the management efficiency improvement of the tourism industry, the energy efficiency of tourism is not necessarily improved. 

**Figure 5:**
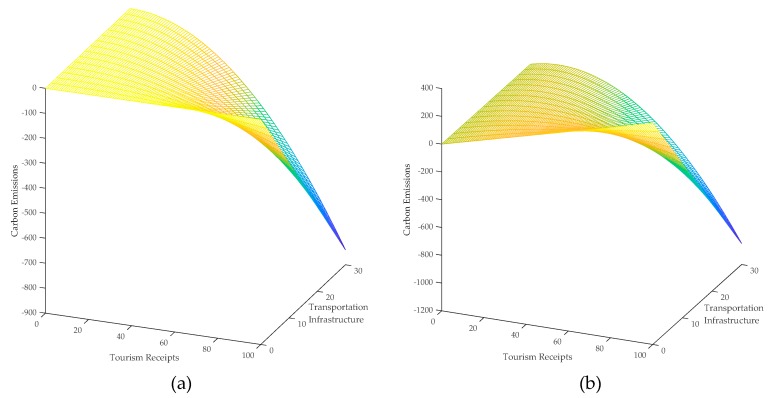
(**a**) Direct and (**b**) indirect effect of tourism on carbon emissions using transportation infrastructure as the moderator.

The results presented in column 8 of Table 4 show that the coefficient of the interaction term of the squared tourism and the moderator is −0.0028 with a significance level of 10%, which indicates that the improvement of the transportation infrastructure is conducive to increasing carbon efficiency and strengthens the negative effect of tourism on carbon emissions. Although the construction of transportation facilities will increase carbon emissions, the improvement of transportation infrastructure will improve the energy efficiency of the transportation industry and thus reduce carbon emissions from tourism [45,46]. Our results show that the aggregated carbon emissions induced by the improvement of transportation infrastructure are significantly negative, and therefore, the improvement of transportation infrastructure has a significant impact on the strength of the relationship between tourism and carbon emissions. Moreover, the coefficient of the spatial lag of the interaction term is −0.0048, with a significance level of 10%, which indicates that the improvement of one province’s transportation infrastructure not only strengthens the negative effect of tourism on its carbon emissions but also strengthens the negative effect of tourism on its neighbors’ carbon emissions. The moderating effects of transportation infrastructure are depicted in Figure 5, from which we can see that with the improvement of transportation infrastructure, both direct and indirect effects of tourism on carbon emissions tend to be strengthened.

## 5. Conclusions and Policy Implications

Considering the possible nonlinear and spatial effects of tourism on carbon emissions, this paper employed spatial econometric models combined with quadratic terms of explanatory variables to explore the nexus between them. The main conclusions of the empirical analysis based on Chinese provincial panel data from 2003 to 2016 are as follows: First, there is a significant inverse U-shaped relationship between tourism development and carbon emissions. In the provinces whose tourism receipts are relatively low, the effects of tourism on carbon emissions are positive but decrease gradually as the tourism receipts increase and then become negative and continue decreasing gradually when the tourism receipts increase beyond the critical value. Second, there is a significant spatial lag effect (indirect effect) of tourism on carbon emissions, and the effect is also inverse U-shaped. One province’s tourism development not only affects its carbon emissions but also affects its neighbors’ carbon emissions because of geographical proximity and industrial relevance. Finally, carbon reduction policy, sustainable education, and transportation infrastructure all have significant moderating effects on the relationship between tourism development and carbon emissions, but the moderating effect of the management efficiency of tourism is not statistically significant. Furthermore, improvement of the sustainable education and transportation infrastructure not only strengthens the direct negative effect of tourism on carbon emissions but also strengthens the indirect negative effect of tourism on carbon emissions.

Although the idea that increasing the emissions of carbon dioxide has a significant effect on global temperatures is not the topic of this study, it is one of the backgrounds of this paper. We noted that the above idea is still controversial. Although the IPCC (Intergovernmental Panel on Climate Change) concluded that increasing carbon dioxide will increase global temperatures [71], some researchers concluded that increases in carbon dioxide emissions have had no significant effects [72,73]. Ignoring the controversy may misinform readers [74]. However, for this article, low-carbon development of tourism will also help to reduce environmental pollution caused by the consumption of fossil fuels and will be conducive to the sustainable development of tourism, because fossil fuels currently still account for roughly 85% of China’s energy mix [75]. Therefore, even considering the uncertainty of the relationship between carbon dioxide and climate change, the study on the nexus between carbon dioxide emissions and tourism is still of great significance and has important policy implications.

Three main policy implications can be drawn from the above conclusions. First, in the regions where tourism receipts are below the critical value (283 billion CNY in China), it is still necessary to improve the carbon efficiency of the tourism industry, because tourism can only be considered as a low carbon industry when its scale exceeds the critical value. Considering the lack of an endogenous driving force to improve carbon efficiency, local government could take measures, such as increasing the investment in green public infrastructure and subsidizing tourism enterprises’ low-carbon initiatives, to improve energy efficiency and the induced carbon efficiency of the tourism industry. Second, local governments could improve the carbon efficiency in the upstream industries of the tourism industry to avoid the positive carbon emissions effect caused by their neighbors’ tourism growth. They could also improve tourism carbon efficiency through strengthening the connectivity between the upstream industry of tourism and the tourism industry of the neighboring regions because of the existing of the negative indirect emissions effect of the tourism industry. Lastly, governments could strengthen the negative effect of tourism on carbon emissions by increasing the number of carbon reduction policies, increasing the years of education, and improving transportation infrastructure. Moreover, tourism enterprises should be encouraged to improve energy efficiency and induced carbon efficiency while improving management efficiency. 

## Figures and Tables

**Table 1:** Description and summary statistics of the variables.

Variable	Description	Unit	Mean	Std. Dev.	Min	Max
Emission	Carbon emissions	million tons	281.90	234.99	7.55	1552.01
Tourism	Tourism receipts	10 billion CNY *	12.56	12.85	0.10	77.39
Energy_con	Energy consumption	10^4^ tons tce **	119.47	78.85	6.84	388.99
Energy_mix	Energy mix	%	68.41	26.18	8.70	98.43
PGDP	GDP per capita	10^3^ CNY	27.81	23.02	3.69	139.34
Reduction	Carbon reduction policy	piece	18.39	20.33	1.00	133.00
Education	Sustainable education	year	8.59	0.99	6.04	12.08
Toueff	Tourism efficiency	10 million CNY per employee	0.17	0.17	0.002	1.01
Trans	Transportation infrastructure	kilometers per 10^2^ square kilometers	4.34	4.83	0.08	26.01

* CNY represents Chinese Yuan, which is a unit of Chinese currency; ** tce means a ton of coal equivalent, which is a unit of energy.

**Table 2:** Moran’s I statistic of carbon emissions from 2003 to 2016.

**Year**	**2003**	**2004**	**2005**	**2006**	**2007**	**2008**	**2009**
Moran’s I	0.17 *	0.18 *	0.19 *	0.16	0.14	0.16	0.17 *
**Year**	**2010**	**2011**	**2012**	**2013**	**2014**	**2015**	**2016**
Moran’s I	0.20*	0.21 **	0.20 *	0.22 **	0.19 *	0.18*	0.17 *

** and * denote *p* < 0.05 and *p* < 0.1, respectively.

**Table 3:** Estimation results of the impact of tourism on carbon emissions.

Model	SDM	SDEM	GNSM	OPM
Tourism	5.7728 *	3.2639 *	5.5860 *	4.5892 **
	(1.82)	(1.73)	(1.75)	(2.40)
Tourism^2^	−0.0678 **	−0.0577 ***	−0.0654 **	−0.0711 ***
	(−2.10)	(−2.58)	(−1.99)	(−3.12)
Energy_con	2.7144	2.2529 ***	2.6729	2.3502 ***
	(1.45)	(8.17)	(1.42)	(8.37)
Energy_mix	−0.0488 **	2.9653 ***	−0.0483 **	3.1082 ***
	(−2.21)	(4.67)	(−2.17)	(5.15)
PGDP	2.3445 ***	−0.9150	2.3631 ***	−0.8199
	(8.82)	(−1.48)	(8.80)	(−1.36)
W*Tourism	2.9775 ***	7.8268 **	3.0156 ***	
	(5.06)	(2.41)	(5.12)	
W*Tourism^2^	−0.8750	−0.0899 ***	−0.8838	
	(−1.51)	(−2.73)	(−1.54)	
W*y	0.2834 ***		0.3030 ***	
	(4.02)		(3.68)	
W*u		0.1802 **	−0.0489	
		(2.03)	(−0.43)	
Log L	−2267.08	−2272.58	−2266.99	
LR test	0.19	11.18 ***		
Province FE	Y	Y	Y	Y
Year FE	Y	Y	Y	Y
N	420	420	420	420
Chi2	633.56 ***	417.72 ***	679.93 ***	-
R-Square	0.80	0.80	0.80	0.54

(1) the *t*-statistic of each coefficient was shown in brackets. (2) *** *p* < 0.01, ** *p* < 0.05, * *p* < 0.1. (3) “FE” represents fixed effect.

**Table 4:** Estimation results of the moderating effects.

Model	SDEM	SEM	SDEM	SEM	SDEM	SEM	SDEM	SEM
(*moderator*)	(*reduction*)	(*reduction*)	(*education*)	(*education*)	(*toueff*)	(*toueff*)	(*trans*)	(*trans*)
Tourism^2^×moderator	−0.0009 **	−0.0007 **	−0.0057 **	−0.0053 **	−0.0285	−0.0192	−0.0028 *	−0.0026
	(−2.34)	(−1.98)	(−2.49)	(−2.19)	(−1.05)	(−0.74)	(−1.80)	(−1.61)
Tourism	1.0644	0.9122	2.7594	2.9170	0.4960	0.2398	0.6066	0.5753
	(0.93)	(0.80)	(1.58)	(1.61)	(0.34)	(0.17)	(0.54)	(0.51)
Energy_con	2.3200 ***	2.3168 ***	2.2640 ***	2.2658 ***	2.2244 ***	2.2197 ***	2.3678 ***	2.3355 ***
	(8.42)	(8.41)	(8.23)	(8.22)	(7.71)	(7.76)	(8.50)	(8.38)
Energy_mix	2.3940 ***	2.3672 ***	2.8948 ***	2.6185 ***	2.5218 ***	2.4027 ***	2.6335 ***	2.4441 ***
	(3.87)	(3.87)	(4.58)	(4.16)	(3.98)	(3.86)	(4.19)	(3.96)
PGDP	−1.0465 *	−1.1319*	−0.8211	−0.9424	−1.2273 **	−1.2998 **	−0.6497	−0.9109
	(−1.68)	(−1.85)	(−1.30)	(−1.51)	(−1.98)	(−2.13)	(−0.97)	(−1.38)
W*Tourism^2^	−0.0009		−0.0091 ***		−0.0507		−0.0048 *	
×moderator	(−1.52)		(−2.75)		(−1.27)		(−1.98)	
W*Tourism	2.5845		7.3268 **		2.0670		3.9163 *	
	(1.19)		(2.40)		(1.03)		(1.72)	
W*u	0.2330 ***	0.2279 ***	0.1874 **	0.1907 **	0.2405 ***	0.2422 ***	0.2084 **	0.2170 **
	(2.76)	(2.68)	(2.13)	(2.07)	(2.88)	(2.90)	(2.40)	(2.47)
Log-L	−2276	−2277	−2273	−2277	−2278	−2279	−2276	−2278
LR test	2.33	-	7.64 **	-	1.62	-	3.88 **	-
Year FE	Y	Y	Y	Y	Y	Y	Y	Y
Province FE	Y	Y	Y	Y	Y	Y	Y	Y
N	420	420	420	420	420	420	420	420
Chi2	363.52	359.94	410.93	386.73	349.95	344.69	381.14	364.69
Pseudo.R-Square	0.80	0.80	0.80	0.80	0.80	0.79	0.80	0.80

(1) the *t*-statistics of each coefficient was shown in brackets. (2) *** *p* < 0.01, ** *p* < 0.05, * *p* < 0.1. (3) “FE” represents fixed effect.
